# A scoping review protocol: Neuropsychological development in pediatric ophthalmology patients with social determinants of health analysis

**DOI:** 10.1371/journal.pone.0330357

**Published:** 2025-08-12

**Authors:** Euna Cho, Justin Zhang, Rebecca Li, Sua Cho, David Kim, Emily Gorman, Moran Roni Levin, Patrice Hicks, Janet Leath Alexander

**Affiliations:** 1 Department of Ophthalmology and Visual Sciences, University of Maryland School of Medicine, Baltimore, Maryland, United States of America; 2 Department of Ophthalmology, University of Rochester School of Medicine and Dentistry, Rochester, New York, United States of America; 3 Department of Psychology, Penn State University, University Park, Pennsylvania, United States of America; 4 Trinity College of Arts and Sciences, Duke University, Durham, North Carolina, United States of America; 5 Health Sciences and Human Services Library, University of Maryland, Baltimore, Maryland, United States of America; 6 Department of Ophthalmology and Visual Sciences, University of Michigan, Ann Arbor, Michigan, United States of America; Aravind Eye Hospital, INDIA

## Abstract

**Introduction:**

Globally, ocular diseases have a substantial prevalence and impose a significant disease burden. Specifically, ocular diseases can negatively impact neuropsychological development for pediatric patients, including academic, social, and mental health as well as quality of life. Neuropsychological development is important, because it impacts cognitive functioning and learning, emotional and behavioral regulation, social interactions and communication, executive functions later in life, and long-term health and well-being. Detriments in neuropsychological development can be modulated by the intricate social determinants of health (SDOH) in a patient’s environment, potentially leading to exacerbated outcomes and disparities. We will conduct a scoping review with the aim of evaluating how SDOH influences the impact of ocular diseases on the neuropsychological development of pediatric patients.

**Methods and analysis:**

Using a comprehensive search strategy, all relevant literature will be extracted from the following electronic databases: Cochrane Central Register of Controlled Trials (WileyOnline interface), Embase (Elsevier interface), Europe PMC, Medline (Ovid Interface), PsycInfo (EBSCO interface), and Scopus (Elsevier interface). Inclusion criteria consist of ocular disease, neuropsychiatric development, pediatric population, and social determinants of health. Covidence (Veritas Health Innovation Ltd, Melbourne, Australia) review software will be used to screen articles that meet the inclusion criteria. Extracted articles will be classified according to national income level and universal healthcare index, type of ocular disease, neuropsychological category, and social determinants of health domain. Data analysis will include a quantitative report on the percentage classification of articles by each ocular, neuropsychological, and SDOH domain category. Sub-categorization of article count by national location, income level, and presence of universal healthcare will be analyzed for each explored and observed SDOH domain.

## Introduction

Ophthalmic disorders represent a major global disease burden. In 2020, it was estimated that 1.1 billion people globally are living with vision impairment; this figure is projected to reach 1.76 billion people by 2050 due to an expanding and aging population [1]. Existing studies have thoroughly investigated how ocular disorders such as strabismus, refractive errors, and congenital blindness are associated with significant morbidity, with those diagnosed being more likely to experience academic deficits, mental health disorders, barriers to social interaction, and decreased self-esteem and quality of life [2]. For example, a study conducted by Astle et al. found that adults with strabismus have decreased self-esteem, fewer social relationships, increased prevalence of mental health disorders, and reduced quality of life [2]. These factors are important because they are interconnected and impact an individual’s well-being and overall functioning; for example, a lack of close relationships is strongly linked to depression [3], poor physical health [4], and even higher mortality [5].

Pediatric populations are particularly impacted by ocular diseases due to infancy, childhood, and adolescent periods being critical windows for development, learning, and social growth [6,7]. Studies by Alvarez-Peregrina et al. and Joseph have identified an association between worse visual health and poor academic performance, specifically that of uncorrected refractive errors hindering education, personality development, and career opportunities [8,9]. Thus, it is important that clinical tests properly identify children at risk of underachieving academically and allow clinicians to provide additional support in managing these ocular disorders in pediatric patients [10]. Additionally, Mojon-Azzi et al. found that children with strabismus are invited to fewer birthday parties, specifically that children over six years old with a visible squint are less likely to be accepted by their peers [11]. It can be inferred from this finding that negative social and cognitive biases towards strabismus emerge around age six. There is an abundance of literature that has investigated the impact of ocular diseases on the academic and social development of children, but it is also necessary to include an analysis of how social determinants of health (SDOH) modify neuropsychological development in these pediatric ocular patients.

The United States Department of Health and Human Services defines SDOH as “the conditions in the environments where people are born, live, learn, work, play, worship, and age that affect a wide range of health, functioning, and quality-of-life outcomes and risks [12].” SDOH can thereby attenuate or exacerbate ocular disease burden, influencing the broad outcomes of these diseases on patients’ lives through a complex interplay between external factors and disease burden. These relationships are exemplified by observations that patients with higher socioeconomic status (SES), better access to quality healthcare and education, better living environments, and/or more supportive communities are better equipped to mitigate the detrimental effects of the disease [13].

While many studies have investigated the impact of ocular diseases on the neuropsychological development of children, these tend to focus primarily on the impact of a specific ocular disease while controlling for SDOH factors to avoid confounding rather than analyzing their potential effect(s) on the association between ocular disease and neuropsychological development [14–16]. Using a scoping review methodology, we plan to investigate the existing literature with an emphasis on the three-way intersectionality between pediatric ophthalmology, neuropsychology, and SDOH.

The concept of a scoping review and its nature as “a form of knowledge synthesis, which incorporates a range of study designs to comprehensively summarize and synthesize evidence” are particularly effective for investigating this complex interaction due to the immense breadth of SDOH factors, ocular diseases, and potential developmental effects that exist in the world [17]. We aim to extract information from a variety of articles analyzing a diverse set of ocular diseases and effects and synthesize this information to inform a complex understanding of how SDOH factors influence the outcomes of pediatric ocular diseases and identify gaps to guide future studies in the growing field of SDOH research. Through a thorough review of the literature, we hope to contribute to a more complete understanding of how diseases do not have definitive outcomes for patients but are instead unique to individual patients based on SDOH factors that they exist within. This information will be crucial for informing and directing future medical and public health efforts to improve the outcomes of our communities.

## Methods

In this scoping review, we will conduct a comprehensive review of the literature in accordance with guidelines delineated by the Joanna Briggs Institute [18] and the Preferred Reporting Items for Systematic Reviews and Meta-Analyses extension for Scoping Reviews (PRISMA-ScR) checklist [19]. The completed scoping review will be presented to address questions concerning (1) the distribution of modern literature among the five defined domains of social determinants of health, (2) the variety of ocular diseases and neuropsychological impacts that have been investigated in the literature, and (3) the distribution of literature among countries of various resource levels. These research objectives will form a framework for our presentation of data, and we will provide summary tables highlighting the distributions we uncovered. The current document serves as a scoping review protocol, and the Preferred Reporting Items for Systematic Reviews and Meta-Analyses Protocol (PRISMA-P) can be found in the supporting information. Institutional review board approval was not required for this study because there were no human subjects recruited or involved.

### Research questions

With SDOH research expanding dramatically in recent years, we want to highlight the current state of SDOH influences in ocular disease progression by addressing the following questions:

1. What ocular diseases, neuropsychological impacts, and SDOH modulating factors have been thoroughly investigated in the literature thus far? And which remain relatively lacking?
2. How does the distribution of research internationally vary with available economic and healthcare resources?

### Study eligibility and exclusion

We will define eligible studies as those that focus on a combination of topics: (1) primarily ocular diseases, (2) how these diseases influence the neuropsychological development of pediatric patients, and (3) how SDOH domains modulate this impact. Papers that cover all three topics will be eligible for inclusion.

As such, we will exclude studies that focus on a broader systemic disease, such as Gaucher disease and mucopolysaccharidosis type-I, of which ocular defects are a symptom or comorbidity. Studies that focus on systemic diseases that have ocular involvement will be excluded due to the potential for mixed effects and confounding, since systemic diseases may themselves have effects on neuropsychological development. Studies that focus on ocular disease prevalence and management without addressing how the disease is impacting the neuropsychological development of pediatric patients or how broader SDOH domains influence the disease will also be excluded. We will exclude case reports, case series, conference abstracts, posters, editorials, letters and commentaries without full-length texts, non-English language articles, and secondary literature such as scoping and systematic reviews. Our search will be limited to English language articles only due to a lack of translation resources for other languages.

### Search strategy and data sources

With the collaboration of a research and education librarian, we developed an initial search strategy that was finalized by review with the rest of the team (S2 File).

Using a comprehensive search strategy, all relevant literature will be extracted from the following electronic databases: Cochrane Central Register of Controlled Trials (WileyOnline interface), Embase (Elsevier interface), Europe PMC, Medline (Ovid Interface), PsycInfo (EBSCO interface), and Scopus (Elsevier interface). Inclusion criteria consist of ocular disease, neuropsychiatric development, pediatric population, and social determinants of health. Covidence review software will be used to screen articles that meet the inclusion criteria [20]. Extracted articles will be classified according to national income level and universal healthcare index, type of ocular disease, neuropsychological category, and social determinants of health domain. Data analysis will include a quantitative report on the percentage classification of articles by each ocular, neuropsychological, and SDOH domain category. Sub-categorization of article count by country of origin, income level, and presence of universal healthcare will be analyzed for each explored and observed SDOH domain.

### Study selection

Retrieved articles will first be screened for eligibility by reviewing their titles and abstracts. Then, the full texts for selected articles will be thoroughly screened for inclusion. Both screening phases will be independently conducted by all team members (EC, JZ, RL, SC, DK). A calibration exercise will be conducted on 10% of the total articles identified, and screening will commence once 75% agreement is reached between the 5 primary reviewers (EC, JZ, RL, SC, and DK). Full-text review will involve retrieving and managing records through EndNote X8 and Zotero before having two team members (EC, JZ, RL, SC, or DK) review all full-text documents, exclude irrelevant texts, and document the reason for exclusion. During these two screening stages, disagreements between two members on inclusion or reason for exclusion will be resolved by discussion or a third reviewer to reach a final consensus. Cohen’s kappa statistic will be used to quantify inter-rater reliability for title and abstract screening and full text review.

### Outcomes and data analysis

The extraction of data from the final set of included articles will be done under the guidance of a data extraction template constructed in Covidence. The following data will be extracted from studies using the data extraction forms:

1. General information, including study ID, title, objectives, and method details (e.g., study inclusion criteria, sample size, and sample descriptors)
2. Country information: country in which the study was conducted, national income level (high, upper-middle, low-middle, low), and universal healthcare index (yes or no). The national income level for each country will be determined based on classification status from the World Bank [21]. The universal healthcare index for each country will be determined based on the World Health Organization’s (WHO) 2023 report tracking universal health coverage [22]. Countries were stratified into countries with and without universal healthcare (UHC) based on their latest service coverage index (SCI) from the 2023 WHO study as a part of sustainable development goals (SDG) analyses. Based on existing literature, a cutoff value of at least 80 for a country’s SCI was used to label the country as having universal healthcare. The threshold of UHC SCI ≥ 80 was based on the published literature on this index, where UHC SCI ≥ 80 defined the highest level of service coverage provision [23].
3. Ocular disease of interest (cataract, strabismus, visual acuity, blindness, other), including notable ocular phrases within the article.
4. Neuropsychological categories (learning and intelligence, mental disorder/health, social interaction/communication, self-identity, quality of life, social and cognitive bias, other), including notable neuropsychological phrases within the article. Specific mental health and learning disorders include conditions listed under “Neurodevelopmental Disorders,” “Depressive Disorders,” and “Anxiety Disorders” of the DSM-5.
5. Social determinants of health (economic security, education access and quality, health care access and quality, neighborhood and built environment, social and community context), including notable SDOH phrases within the article [12]. Examples of keywords that can indicate the presence of SDOH domain analysis in the text:
  - Economic security: “income,” “socioeconomic status”
  - Education access and quality: “special needs schools,” “trained teachers”
  - Healthcare access and quality: “access to an ophthalmologist,” “frequency of follow-up”
  - Neighborhood and built environment: “parks,” “recreational spaces,” “pollution”
  - Social and community context: “peers,” “prejudice,” “childhood environment”

Excerpts and quotes pertaining to ocular conditions, neuropsychological impact, and SDOH domains will be included in the data extraction templates to aid with discussion and consensus among the reviewers. For each study, two team members (EC, JZ, RL, SC, or DK) will complete the data extraction template, and discrepancies will be resolved by discussion among the reviewers with a third reviewer finalizing a consensus of the data extraction form.

The final data collected through the data extraction sheets will be converted into tables that show the distribution of research products with regard to the investigated ocular diseases, neuropsychological impacts, and SDOH domains. We will also compile a table demonstrating the distribution of research products across their country of origin and that country’s income levels and UHC status. We will conclude by discussing the data that we have synthesized and identifying which diseases, developmental impacts, and SDOH domains have been thoroughly investigated and which require more research efforts.

### Expected study timeline

This study protocol is ongoing and is expected to be completed by the following timelines (presented in DD/MM/YYYY format):

01/06/2025: Record screening completion

01/08/2025: Data extraction completion

01/09/2025: Results completion

### Ethics and dissemination

The results of the finalized scoping review will be reported in peer-reviewed publications. The identified gaps in the literature will highlight key domains in which resources may be distributed accordingly to promote neuropsychological child development in pediatric patients with ophthalmic diseases.

## Discussion

Understanding how SDOH modulates the developmental impact of disease processes on children is a growing body of research that is essential for the development of effective public health interventions to address these factors beyond the control of patients and their healthcare providers. This scoping review will investigate the existing medical literature with emphasis on the three-way interactions between ophthalmology, neuropsychology, and SDOH components. In contrast to the previous articles focusing on direct correlations between ocular diseases and neuropsychology, this scoping review will provide a comprehensive analysis of whether these articles address SDOH. The three main components of our results will consist of the following. First, the percentage of articles classified into each ocular, neuropsychological, and SDOH categories will be reported. For each of the five SDOH domains, the quantity and percentage of articles exploring and observing the domains will be reported with sub-classification by domestic or international location, national income levels, and presence of UHC. The comparison between explored and observed SDOH domains will provide insight into finding gaps in the literature and suggest where potential intervention may take place. This identification may guide the effective distribution of resources in promoting education and social awareness to reduce stigma and protect child development within individual and community contexts, while tailored policies and interventions are implemented at organizational and systemic levels. The results of the finalized scoping review will be reported in peer-reviewed publications.

As the influence of SDOH domains is a concept that has garnered research interest in recent years, we foresee potential difficulties with retrieving older articles that are relevant to this scoping review and identifying the SDOH domains involved, as they may not be explicitly defined. We believe that this should not significantly reduce the volume of research that can be included in this scoping review below what we could retrieve in an ideal scenario. As such, this shortcoming should not dramatically modify our findings pertaining to the relevant interest in research into each SDOH domain or future interventional efforts.

Overall, we believe that this scoping review will help identify well-informed areas of SDOH research pertaining to pediatric ophthalmic conditions as well as evidence gaps that can be explored through future research efforts. We hope to use the information gleaned from this review to initiate future research projects and initiatives that can fill in identified gaps in the literature and help spur the development of relevant public health interventions.

**Strengths and limitations of this study**

- This scoping review will address the SDOH context in the medical literature on how ophthalmic conditions in pediatric patients affect their neuropsychological development.
- Both U.S. and international literature will be included with sub-analysis on national income level and universal healthcare (UHC) providing additional SDOH context.
- As SDOH is a fairly new area of research in pediatric ophthalmology, we acknowledge that previous literature may not directly mention SDOH domains.

## Supporting information

S1 File Contains PRISMA-P (Preferred Reporting Items for Systematic review and Meta-Analysis Protocols) 2015 checklist. This is the completed PRISMA-P checklist for this protocol.
(DOCX)

S2 File Contains search terms for recruiting papers from databases. (DOCX)
