# Supplementary material for: A scoping review protocol: Neuropsychological development in pediatric ophthalmology patients with social determinants of health analysis
Source: PLoS One. 2025 Aug 12;20(8):e0330357. doi: 10.1371/journal.pone.0330357 (PMC12342284; doi:10.1371/journal.pone.0330357)
Supplement: S2 File — (DOCX) [file pone.0330357.s002.docx]

**Appendix - Scoping Review Search Strategies**

Search developed by Emily F. Gorman, MLIS and reviewed by Nicole Shelawala, M. Eng., MLIS

Filters / Limits: none

**Embase (Elsevier)**

*One-line search run in the Quick search tab of the Embase.com interface:*

('eye disease'/exp/mj OR 'eye surgery'/exp/mj OR 'visually impaired person'/exp/mj OR 'ophthalmology'/exp/mj OR (((eye OR ocular OR oculomotor OR vision OR visual OR ophthalmic OR lens OR retina* OR cornea* OR pupil OR sclera* OR uvea* OR vitreous) NEXT/2 (health OR disease* OR impair* OR disorder* OR abnormal* OR disturbance* OR disab* OR loss)):ti,ab,kw) OR stabism*:ti,ab,kw OR squint:ti,ab,kw OR hypertropia*:ti,ab,kw OR amblyop*:ti,ab,kw OR cataract*:ti,ab,kw OR 'lens clouding':ti,ab,kw OR 'lens opacity':ti,ab,kw OR blindness:ti,ab,kw OR amauros*:ti,ab,kw OR 'low vision':ti,ab,kw OR ((retina* NEAR/1 detach*):ti,ab,kw) OR glaucoma:ti,ab,kw OR ((macula* NEAR/2 (degeneration OR atrophy OR dystrophy)):ti,ab,kw) OR maculopath*:ti,ab,kw OR myopi*:ti,ab,kw OR nearsighted*:ti,ab,kw OR 'near sighted*':ti,ab,kw OR oculopathy:ti,ab,kw OR ophthalmo*:ti,ab,kw OR 'refraction error*':ti,ab,kw OR astigmatism:ti,ab,kw OR hyperop*:ti,ab,kw OR farsighted*:ti,ab,kw OR 'far sighted*':ti,ab,kw OR longsighted*:ti,ab,kw OR 'long sighted*':ti,ab,kw OR macropsia*:ti,ab,kw OR micropsia*:ti,ab,kw OR 'loss of vision':ti,ab,kw) AND ('child'/exp OR 'childhood'/exp OR 'pediatric'/exp OR 'child health'/exp OR 'adolescent'/exp OR 'adolescence'/exp OR 'newborn period'/exp OR child*:ti,ab,kw OR pediatric*:ti,ab,kw OR paediatric*:ti,ab,kw OR pediatry:ti,ab,kw OR adolescen*:ti,ab,kw OR teen*:ti,ab,kw OR youth*:ti,ab,kw) AND ('neuropsychological development'/exp OR 'psychological development'/exp OR 'mental disease'/exp OR 'mental stress'/exp OR 'child psychiatry'/exp OR 'child psychology'/exp OR 'childhood psychosis'/exp OR 'neuropsychological assessment'/exp OR 'behavior disorder'/exp OR 'attitude'/exp OR 'perception'/de OR 'social desirability'/exp OR 'self concept'/exp OR 'academic achievement'/exp OR psychologic*:ti,ab,kw OR neuropsych*:ti,ab,kw OR psychiatr*:ti,ab,kw OR psychopathology:ti,ab,kw OR ((mental NEXT/1 (health OR illness* OR disease* OR disorder* OR stress* OR defect* OR instability)):ti,ab,kw) OR 'mentally ill':ti,ab,kw OR 'attention deficit':ti,ab,kw OR adhd:ti,ab,kw OR (((behavior* OR behaviour*) NEAR/2 (disorder* OR disturbance)):ti,ab,kw) OR (((academic OR school) NEAR/2 (performance OR achievement*)):ti,ab,kw) OR (((social* OR peer*) NEAR/2 (accept* OR wellbeing OR 'well being' OR interact*)):ti,ab,kw) OR perception*:ti,ab,kw OR attitude*:ti,ab,kw OR ((self NEXT/1 (concept OR esteem OR confidence OR image)):ti,ab,kw) OR 'body image':ti,ab,kw) AND ('social determinants of health'/exp OR 'health care access'/exp OR 'health insurance'/exp OR 'health literacy'/exp OR 'educational attainment'/exp OR 'civic engagement'/exp OR 'community engagement'/exp OR 'community participation'/exp OR 'social participation'/exp OR 'social inclusion'/exp OR 'integration'/exp OR 'sense of community'/exp OR 'sense of belonging'/exp OR 'isolation'/exp OR 'loneliness'/exp OR 'social support'/exp OR 'social network'/exp OR 'prejudice'/exp OR 'social discrimination'/exp OR 'workplace'/exp OR 'incarceration'/exp OR 'prisoner'/exp OR 'economic status'/exp OR 'income'/exp OR 'financial security'/exp OR 'cost of living'/exp OR 'socioeconomics'/exp OR 'social status'/exp OR 'social protection'/exp OR 'social environment'/exp OR 'public policy'/exp OR 'employment'/exp OR 'unemployment'/exp OR 'food security'/exp OR 'housing'/exp OR 'crime'/exp OR 'violence'/exp OR 'physical environment'/exp OR 'traffic and transport'/exp OR 'air quality'/exp OR 'air pollution'/exp OR 'water quality'/exp OR 'healthy food'/exp OR 'food desert'/exp OR 'communication barrier'/exp OR 'cultural anthropology'/exp OR 'physiological stress'/exp OR 'mental stress'/exp OR 'physical activity'/exp OR 'exercise'/exp OR 'recreational park'/exp OR 'walkability'/exp OR 'playground'/exp OR 'social determin*':ti,ab,kw OR 'social factor*':ti,ab,kw OR 'social health determinant*':ti,ab,kw OR sdoh:ti,ab,kw OR 'health structural determinant*':ti,ab,kw OR (((healthcare OR 'care' OR 'health services') NEAR/2 access*):ti,ab,kw) OR (((health OR healthcare OR 'health care' OR medical OR sickness) NEXT/1 (insurance OR benefit*)):ti,ab,kw) OR 'health literacy':ti,ab,kw OR 'ehealth literacy':ti,ab,kw OR 'education level*':ti,ab,kw OR 'level of education':ti,ab,kw OR 'educational attainment':ti,ab,kw OR 'educational achievement':ti,ab,kw OR (((civic OR social OR community OR public) NEXT/1 (participation OR engagement OR integration OR inclusion OR involvement)):ti,ab,kw) OR 'sense of belonging':ti,ab,kw OR 'sense of community':ti,ab,kw OR isolation:ti,ab,kw OR loneliness:ti,ab,kw OR discrimination:ti,ab,kw OR prejudice*:ti,ab,kw OR racism:ti,ab,kw OR homophobia:ti,ab,kw OR biphobia:ti,ab,kw OR transphobia:ti,ab,kw OR 'workplace condition*':ti,ab,kw OR 'workplace environment':ti,ab,kw OR incarceration:ti,ab,kw OR imprisonment:ti,ab,kw OR prisoner*:ti,ab,kw OR 'economic stability':ti,ab,kw OR income:ti,ab,kw OR 'cost of living':ti,ab,kw OR socioeconomic*:ti,ab,kw OR 'socio-economic*':ti,ab,kw OR 'social economic status':ti,ab,kw OR ((social NEXT/1 (status OR achievement OR condition* OR protection OR polic* OR environment OR connectedness OR cohesion OR context* OR exclu* OR support* OR network*)):ti,ab,kw) OR 'public polic*':ti,ab,kw OR 'government* polic*':ti,ab,kw OR poverty:ti,ab,kw OR employment:ti,ab,kw OR unemployment:ti,ab,kw OR 'occupational status':ti,ab,kw OR 'job security':ti,ab,kw OR 'job insecurity':ti,ab,kw OR 'food security':ti,ab,kw OR 'food insecurity':ti,ab,kw OR housing:ti,ab,kw OR lodging*:ti,ab,kw OR 'living arrangement*':ti,ab,kw OR transportation:ti,ab,kw OR 'air quality':ti,ab,kw OR 'air pollution':ti,ab,kw OR 'water quality':ti,ab,kw OR 'healthy food*':ti,ab,kw OR 'food desert*':ti,ab,kw OR neighborhood*:ti,ab,kw OR neighbourhood*:ti,ab,kw OR 'physical environment':ti,ab,kw OR crime:ti,ab,kw OR violence:ti,ab,kw OR 'language barrier*':ti,ab,kw OR 'communication barrier*':ti,ab,kw OR (driver* NEXT/2 health):ti,ab,kw OR doh:ti,ab,kw OR 'structural conflict*':ti,ab,kw OR culture:ti,ab,kw OR stress:ti,ab,kw OR 'physical activit*':ti,ab,kw OR exercise:ti,ab,kw OR parks:ti,ab,kw OR 'green space*':ti,ab,kw OR walkability:ti,ab,kw OR 'walk friendly':ti,ab,kw OR 'walking friendly':ti,ab,kw OR playground*:ti,ab,kw)

**MEDLINE ALL (Ovid)**

*Multi-line search run in the Advanced Search interface:*

1. exp *Eye Diseases/ or exp *visually impaired persons/ or exp *cataract/ or exp *glaucoma/ or exp *ophthalmologic surgical procedures/ or exp *ophthalmology/ or exp *retinal degeneration/
2. (((eye or ocular or oculomotor or vision or visual or ophthalmic or lens or retina* or cornea* or pupil or sclera* or uvea* or vitreous) adj2 (health or disease* or impair* or disorder* or abnormal* or disturbance* or disab* or loss)) or stabism* or squint or hypertropia* or amblyop* or cataract* or lens clouding or lens opacity or blindness or amauros* or low vision or (retina* adj detach*) or glaucoma or (macula* adj2 (degeneration or atrophy or dystrophy)) or maculopath* or myopi* or nearsighted* or near sighted* or oculopathy or ophthalmo* or refraction error* or astigmatism or hyperop* or farsighted* or far sighted* or longsighted* or long sighted* or macropsia* or micropsia* or "loss of vision").ti,ab,kw.
3. 1 or 2
4. exp adolescent/ or exp child/ or exp infant/ or exp adolescence/ or exp minors/
5. (child* or pediatric* or paediatric* or pediatry or adolescen* or teen* or youth*).ti,ab,kw.
6. 4 or 5
7. child development/ or exp neuropsychological tests/ or exp mental disorders/ or exp "stress, psychological"/ or exp child psychiatry/ or exp "psychology, child"/ or exp "psychology, developmental"/ or exp "psychology, adolescent"/ or exp neurodevelopmental disorders/ or exp social perception/ or exp attitude/ or exp self concept/ or exp social desirability/ or exp academic performance/
8. (psychologic* or neuropsych* or psychiatr* or psychopathology or (mental adj (health or illness* or disease* or disorder* or stress* or defect* or instability)) or mentally ill or attention deficit or adhd or ((behavior* or behaviour*) adj2 (disorder* or disturbance)) or ((academic or school) adj2 (performance or achievement*)) or ((social* or peer*) adj2 (accept* or wellbeing or well being or interact*)) or perception* or attitude* or (self adj (concept or esteem or confidence or image)) or body image).ti,ab,kw.
9. 7 or 8
10. exp social determinants of health/ or exp health services accessibility/ or exp insurance, health/ or exp health literacy/ or exp educational status/ or exp social participation/ or exp community participation/ or exp social inclusion/ or exp social integration/ or exp social isolation/ or exp social support/ or exp social networking/ or exp prejudice/ or exp social discrimination/ or exp workplace/ or exp prisoners/ or exp economic stability/ or exp income/ or exp economics/ or exp socioeconomic factors/ or exp public policy/ or exp social environment/ or exp food security/ or exp food supply/ or exp housing/ or exp residence characteristics/ or exp crime/ or exp violence/ or exp transportation/ or exp air pollution/ or exp water quality/ or exp food deserts/ or exp communication barriers/ or exp culture/ or exp stress, psychological/ or exp exercise/ or exp parks, recreational/
11. (social determin* or social factor* or social health determinant* or sdoh or health structural determinant* or ((healthcare or care or health services) adj2 access*) or ((health or healthcare or health care or medical or sickness) adj (insurance or benefit*)) or health literacy or ehealth literacy or education level* or level of education or educational attainment or educational achievement or ((civic or social or community or public) adj (participation or engagement or integration or inclusion or involvement)) or "sense of belonging" or "sense of community" or isolation or loneliness or discrimination or prejudice* or racism or homophobia or biphobia or transphobia or workplace condition* or workplace environment or incarceration or imprisonment or prisoner* or economic stability or income or "cost of living" or socioeconomic* or socio-economic* or social economic status or (social adj (status or achievement or condition* or protection or polic* or environment or connectedness or cohesion or context* or exclu* or support* or network*)) or public polic* or government* polic* or poverty or employment or unemployment or occupational status or job security or job insecurity or food security or food insecurity or housing or lodging* or living arrangement* or transportation or air quality or air pollution or water quality or healthy food* or food desert* or neighborhood* or neighbourhood* or physical environment or crime or violence or language barrier* or communication barrier* or (driver* adj2 health) or doh or structural conflict* or culture or stress or physical activit* or exercise or parks or green space* or walkability or walk friendly or walking friendly or playground*).ti,ab,kw.
12. 10 or 11
13. 3 and 6 and 9 and 12

**Cochrane Central Register of Controlled Trials (WileyOnline)**

*Multi-line search run in the Search Manager interface of Advanced Search:*

1. [mh "Eye Diseases"[mj]] or [mh "visually impaired persons"[mj]] or [mh cataract[mj]] or [mh glaucoma[mj]] or [mh "ophthalmologic surgical procedures"[mj]] or [mh ophthalmology[mj]] or [mh "retinal degeneration"[mj]]
2. (((eye or ocular or oculomotor or vision or visual or ophthalmic or lens or retina* or cornea* or pupil or sclera* or uvea* or vitreous) next/2 (health or disease* or impair* or disorder* or abnormal* or disturbance* or disab* or loss)) or stabism* or squint or hypertropia* or amblyop* or cataract* or "lens clouding" or "lens opacity" or blindness or amauros* or "low vision" or (retina* near detach*) or glaucoma or (macula* near/2 (degeneration or atrophy or dystrophy)) or maculopath* or myopi* or nearsighted* or ("near" next sighted*) or oculopathy or ophthalmo* or (refraction next error*) or astigmatism or hyperop* or farsighted* or (far next sighted*) or longsighted* or (long next sighted*) or macropsia* or micropsia* or "loss of vision"):ti,ab,kw
3. #1 or #2
4. [mh adolescent] or [mh child] or [mh infant] or [mh adolescence] or [mh minors]
5. (child* or pediatric* or paediatric* or pediatry or adolescen* or teen* or youth*):ti,ab,kw
6. #4 or #5
7. [mh ^"child development"] or [mh "neuropsychological tests"] or [mh "mental disorders"] or [mh "stress, psychological"] or [mh "child psychiatry"] or [mh "psychology, child"] or [mh "psychology, developmental"] or [mh "psychology, adolescent"] or [mh "neurodevelopmental disorders"] or [mh "social perception"] or [mh attitude] or [mh "self concept"] or [mh "social desirability"] or [mh "academic performance"]
8. (psychologic* or neuropsych* or psychiatr* or psychopathology or (mental next (health or illness* or disease* or disorder* or stress* or defect* or instability)) or "mentally ill" or "attention deficit" or adhd or ((behavior* or behaviour*) near/2 (disorder* or disturbance)) or ((academic OR school) near/2 (performance OR achievement*)) or ((social* or peer*) near/2 (accept* or wellbeing or well being or interact*)) or perception* or attitude* or (self next (concept or esteem or confidence or image)) or "body image"):ti,ab,kw
9. #7 or #8
10. [mh "social determinants of health"] or [mh "health services accessibility"] or [mh "insurance, health"] or [mh "health literacy"] or [mh "educational status"] or [mh "social participation"] or [mh "community participation"] or [mh "social discrimination"] or [mh prejudice] or [mh workplace] or [mh prisoners] or [mh "economic stability"] or [mh income] or [mh economics] or [mh "socioeconomic factors"] or [mh "public policy"] or [mh "social inclusion"] or [mh "social integration"] or [mh "social isolation"] or [mh "social support"] or [mh "social networking"] or [mh "social environment"] or [mh "food security"] or [mh "food supply"] or [mh housing] or [mh "residence characteristics"] or [mh crime] or [mh violence] or [mh transportation] or [mh "air pollution"] or [mh "water quality"] or [mh "food deserts"] or [mh "communication barriers"] or [mh culture] or [mh "stress, psychological"] or [mh exercise] or [mh "parks, recreational"]
11. ((social NEXT determin*) or (social NEXT factor*) or ("social health" NEXT determinant*) or sdoh or ("health structural" NEXT determinant*) or ((healthcare or care or "health services") near/2 access*) or ((health or healthcare or "health care" or medical or sickness) next (insurance or benefit*)) or "health literacy" or "ehealth literacy" or (education NEXT level*) or "level of education" or "educational attainment" or "educational achievement" or ((civic or social or community or public) next (participation or engagement or integration or inclusion or involvement)) or "sense of belonging" or isolation or loneliness or discrimination or prejudice* or "sense of community" or racism or homophobia or biphobia or transphobia or (workplace next condition*) or "workplace environment" or incarceration or imprisonment or prisoner* or "economic stability" or income or "cost of living" or socioeconomic* or socio-economic* or "social economic status" or (social next (status or achievement or condition* or protection or polic* or environment or connectedness or cohesion or context* or exclu* or support* or network*)) or (public NEXT polic*) or (government* NEXT polic*) or poverty or employment or unemployment or "occupational status" or "job security" or "job insecurity" or "food security" or "food insecurity" or housing or lodging* or (living NEXT arrangement*) or transportation or "air quality" or "air pollution" or "water quality" or (healthy NEXT food*) or (food NEXT desert*) or neighborhood* or neighbourhood* or "physical environment" or crime or violence or (language NEXT barrier*) or (communication NEXT barrier*) or (driver* next/2 health) or doh or (structural NEXT conflict*) or culture or stress or (physical NEXT activit*) or exercise or parks or (green NEXT space*) or walkability or "walk friendly" or "walking friendly" or playground*):ti,ab,kw
12. #10 or #11
13. #3 and #6 and #9 and #12

**Scopus (Elsevier)**

*One-line search run in the Advanced Document Search interface:*

TITLE-ABS-KEY ( ( ( eye OR ocular OR oculomotor OR vision OR visual OR ophthalmic OR lens OR retina* OR cornea* OR pupil OR sclera* OR uvea* OR vitreous ) PRE/1 ( health OR disease* OR impair* OR disorder* OR abnormal* OR disturbance* OR disab* OR loss ) ) OR stabism* OR squint OR hypertropia* OR amblyop* OR cataract* OR {lens clouding} OR {lens opacity} OR blindness OR amauros* OR {low vision} OR ( retina* W/0 detach* ) OR glaucoma OR ( macula* W/1 ( degeneration OR atrophy OR dystrophy ) ) OR maculopath* OR myopi* OR nearsighted* OR "near sighted*" OR oculopathy OR ophthalmo* OR "refraction error*" OR astigmatism OR hyperop* OR farsighted* OR "far sighted*" OR longsighted* OR "long sighted*" OR macropsia* OR micropsia* OR {loss of vision} ) AND TITLE-ABS-KEY ( child* OR pediatric* OR paediatric* OR pediatry OR adolescen* OR teen* OR youth* ) AND TITLE-ABS-KEY ( psychologic* OR neuropsych* OR psychiatr* OR psychopathology OR ( mental PRE/0 ( health OR illness* OR disease* OR disorder* OR stress* OR defect* OR instability ) ) OR {mentally ill} OR {attention deficit} OR adhd OR ( ( behavior* OR behaviour* ) W/1 ( disorder* OR disturbance ) ) OR ( ( academic OR school ) W/1 ( performance OR achievement* ) ) OR ( ( social* OR peer* ) W/1 ( accept* OR wellbeing OR "well being" OR interact* ) ) OR perception* OR attitude* OR ( self PRE/0 ( concept OR esteem OR confidence OR image ) ) OR {body image} ) AND TITLE-ABS-KEY ( "social determin*" OR "social factor*" OR "social health determinant*" OR sdoh OR "health structural determinant*" OR ( ( healthcare OR care OR "health services" ) W/1 access* ) OR ( ( health OR healthcare OR "health care" OR medical OR sickness ) PRE/0 ( insurance OR benefit* ) ) OR {health literacy} OR {ehealth literacy} OR "education level*" OR {level of education} OR {educational attainment} OR {educational achievement} OR ( ( civic OR social OR community OR public ) PRE/0 ( participation OR engagement OR integration OR inclusion OR involvement ) ) OR {sense of belonging} OR {sense of community} OR isolation OR loneliness OR discrimination OR prejudice* OR racism OR homophobia OR biphobia OR transphobia OR "workplace condition*" OR {workplace environment} OR incarceration OR imprisonment OR prisoner* OR {economic stability} OR income OR {cost of living} OR socioeconomic* OR socio-economic* OR {social economic status} OR ( social PRE/0 ( status OR achievement OR condition* OR protection OR polic* OR environment OR connectedness OR cohesion OR context* OR exclu* OR support* OR network* ) ) OR "public polic*" OR ( government* PRE/0 polic* ) OR poverty OR employment OR unemployment OR {occupational status} OR {job security} OR {job insecurity} OR {food security} OR {food insecurity} OR housing OR lodging* OR "living arrangement*" OR transportation OR {air quality} OR {air pollution} OR {water quality} OR "healthy food*" OR "food desert*" OR neighborhood* OR neighbourhood* OR {physical environment} OR crime OR violence OR "language barrier*" OR "communication barrier*" OR ( driver* PRE/1 health ) OR doh OR "structural conflict*" OR culture OR stress OR "physical activit*" OR exercise OR parks OR "green space*" OR walkability OR {walk friendly} OR {walking friendly} OR playground* )

**PsycInfo (EBSCO)**

*Multi-line search run in the Advanced Search interface:*

1. (MM "Vision Disorders" OR MM "Balint's Syndrome" OR MM "Blindness" OR MM "Blindsight" OR MM "Eye Disorders" OR MM "Hemianopia" OR MM "Low Vision") OR (MM "Ophthalmology")
2. (AB ( (eye OR ocular OR oculomotor OR vision OR visual OR ophthalmic OR lens OR retina* OR cornea* OR pupil OR sclera* OR uvea* OR vitreous) W2 (health OR disease* OR impair* OR disorder* OR abnormal* OR disturbance* OR disab* OR loss) ) OR AB ( stabism* OR squint OR hypertropia* OR amblyop* OR cataract* OR “lens clouding” OR “lens opacity” OR blindness OR amauros* OR “low vision” OR glaucoma* OR maculopath* OR myopi* OR nearsighted* OR “near sighted*” OR oculopathy OR ophthalmo* OR “refraction error*” OR astigmatism OR hyperop* OR farsighted* OR “far sighted*” OR longsighted* OR “long sighted*” OR macropsia* OR micropsia* OR “loss of vision” ) OR AB retina* N1 detach* OR AB ( macula* N2 (degeneration OR atrophy OR dystrophy) )) OR (TI ( (eye OR ocular OR oculomotor OR vision OR visual OR ophthalmic OR lens OR retina* OR cornea* OR pupil OR sclera* OR uvea* OR vitreous) W2 (health OR disease* OR impair* OR disorder* OR abnormal* OR disturbance* OR disab* OR loss) ) OR TI ( stabism* OR squint OR hypertropia* OR amblyop* OR cataract* OR “lens clouding” OR “lens opacity” OR blindness OR amauros* OR “low vision” OR glaucoma* OR maculopath* OR myopi* OR nearsighted* OR “near sighted*” OR oculopathy OR ophthalmo* OR “refraction error*” OR astigmatism OR hyperop* OR farsighted* OR “far sighted*” OR longsighted* OR “long sighted*” OR macropsia* OR micropsia* OR “loss of vision” ) OR TI retina* N1 detach* OR TI ( macula* N2 (degeneration OR atrophy OR dystrophy) ))
3. S1 OR S2
4. ((((((((DE "Early Adolescence") OR (DE "Late Adolescence")) OR (DE "Postnatal Period")) OR (DE "Neonatal Period")) OR (DE "Puberty")) OR (DE "Child Health")) OR (DE "Child Characteristics")) OR (DE "Adolescent Characteristics")) OR (DE "Adolescent Health")
5. TI (child* OR pediatric* OR paediatric* OR pediatry OR adolescen* OR teen* OR youth*) OR AB (child* OR pediatric* OR paediatric* OR pediatry OR adolescen* OR teen* OR youth*) OR SU (child* OR pediatric* OR paediatric* OR pediatry OR adolescen* OR teen* OR youth*)
6. S4 OR S5
7. ((((((((((((((((((DE "Childhood Development") OR (DE "Child Psychiatry")) OR (DE "Child Psychology")) OR (DE "Child Psychopathology")) OR (DE "Adolescent Psychiatry" OR DE "Adolescent Psychology" OR DE "Adolescent Psychopathology")) OR (DE "Youth Mental Health")) OR (DE "Mental Health")) OR (DE "Mental Disorders" OR DE "Affective Disorders" OR DE "Anxiety Disorders" OR DE "Behavior Disorders" OR DE "Bipolar Disorder" OR DE "Borderline States" OR DE "Chronic Mental Illness" OR DE "Dissociative Disorders" OR DE "Eating Disorders" OR DE "Gender Dysphoria" OR DE "Mental Disorders due to General Medical Conditions" OR DE "Neurocognitive Disorders" OR DE "Neurodevelopmental Disorders" OR DE "Neurosis" OR DE "Obsessive Compulsive Disorder" OR DE "Paraphilias" OR DE "Personality Disorders" OR DE "Psychosis" OR DE "Serious Mental Illness" OR DE "Sleep Wake Disorders" OR DE "Somatoform Disorders" OR DE "Stress and Trauma Related Disorders" OR DE "Substance Related and Addictive Disorders" OR DE "Thought Disorders")) OR (DE "Childhood Onset Psychosis" OR DE "Childhood Onset Schizophrenia")) OR (DE "Neurodevelopmental Disorders" OR DE "Attention Deficit Disorder" OR DE "Autism Spectrum Disorders" OR DE "Communication Disorders" OR DE "Developmental Disabilities" OR DE "Disruptive Behavior Disorders" OR DE "Dyspraxia" OR DE "Emotional and Behavioral Disorders" OR DE "Intellectual Development Disorder" OR DE "Learning Disorders" OR DE "Stereotypic Movement Disorder" OR DE "Tic Disorders")) OR (DE "Neuropsychological Assessment" OR DE "Halstead Reitan Neuropsychological Battery" OR DE "Luria Nebraska Neuropsychological Battery" OR DE "Mini Mental State Examination" OR DE "Task Switching" OR DE "Wisconsin Card Sorting Test")) OR (DE "Psychological Development" OR DE "Cognitive Development" OR DE "Emotional Development" OR DE "Moral Development" OR DE "Nature Nurture" OR DE "Psychosocial Development" OR DE "Socioemotional Functioning")) OR (DE "Attitudes" OR DE "Abortion (Attitudes Toward)" OR DE "Adolescent Attitudes" OR DE "Adult Attitudes" OR DE "Aged (Attitudes Toward)" OR DE "Aging (Attitudes Toward)" OR DE "Attitude Change" OR DE "Attitude Formation" OR DE "Attitude Similarity" OR DE "Caregiver Attitudes" OR DE "Child Attitudes" OR DE "Childrearing Attitudes" OR DE "Client Attitudes" OR DE "Community Attitudes" OR DE "Computer Attitudes" OR DE "Consumer Attitudes" OR DE "Cultural Attitudes" OR DE "Death Attitudes" OR DE "Disabled (Attitudes Toward)" OR DE "Drug Usage Attitudes" OR DE "Eating Attitudes" OR DE "Employee Attitudes" OR DE "Employer Attitudes" OR DE "Environmental Attitudes" OR DE "Explicit Attitudes" OR DE "Family Planning Attitudes" OR DE "Female Attitudes" OR DE "Gender Role Attitudes" OR DE "Health Attitudes" OR DE "Ideology" OR DE "Immigration (Attitudes Toward)" OR DE "Implicit Attitudes" OR DE "Male Attitudes" OR DE "Marriage Attitudes" OR DE "Obesity (Attitudes Toward)" OR DE "Occupational Attitudes" OR DE "Parental Attitudes" OR DE "Paternalism" OR DE "Political Attitudes" OR DE "Preferences" OR DE "Public Opinion" OR DE "Racial and Ethnic Attitudes" OR DE "Sex Role Attitudes" OR DE "Sexual Attitudes" OR DE "Socioeconomic Class Attitudes" OR DE "Sports (Attitudes Toward)" OR DE "Stereotyped Attitudes" OR DE "Student Attitudes" OR DE "Values" OR DE "Work (Attitudes Toward)" OR DE "World View")) OR (DE "Social Perception" OR DE "Anonymity" OR DE "Attribution" OR DE "Credibility" OR DE "Fame" OR DE "Halo Effect" OR DE "Impression Formation" OR DE "Impression Management" OR DE "Labeling" OR DE "Objectification" OR DE "Popularity" OR DE "Reputation" OR DE "Social Comparison" OR DE "Stigma")) OR (DE "Social Desirability")) OR (DE "Self-Concept" OR DE "Academic Self Concept" OR DE "Athletic Identity" OR DE "Entitlement (Psychological)" OR DE "Impostor Phenomenon" OR DE "Professional Identity" OR DE "Self-Affirmation" OR DE "Self-Compassion" OR DE "Self-Confidence" OR DE "Self-Congruence" OR DE "Self-Esteem" OR DE "Self-Forgiveness" OR DE "Self-Regard" OR DE "Self-Worth" OR DE "Sense of Coherence")) OR (DE "Self-Perception" OR DE "Belonging" OR DE "Body Image" OR DE "Interoception" OR DE "Perceived Control" OR DE "Self-Acceptance" OR DE "Self-Deception" OR DE "Self-Efficacy" OR DE "Self-Knowledge" OR DE "Self-Reference" OR DE "Self-Reflection")) OR (DE "Educational Diagnosis")) OR (DE "Academic Achievement" OR DE "Academic Overachievement" OR DE "Academic Underachievement" OR DE "Achievement Gap" OR DE "College Academic Achievement" OR DE "Mathematics Achievement" OR DE "Reading Achievement" OR DE "Science Achievement")
8. (TI ( psychologic* OR neuropsych* OR psychiatr* OR psychopathology OR “mentally ill” OR “attention deficit” OR adhd OR perception* OR attitude* OR "body image" ) OR TI ( mental W1 (health OR illness* OR disease* OR disorder* OR stress* OR defect* OR instability) ) OR TI ( (behavior* OR behaviour*) N2 (disorder* OR disturbance) ) OR TI ( (academic OR school) N2 (performance OR achievement*) ) OR TI ( (social* OR peer*) N2 (accept* OR wellbeing OR "well being" OR interact*) ) OR TI ( self W1 (concept OR esteem OR confidence OR image) )) OR (AB ( psychologic* OR neuropsych* OR psychiatr* OR psychopathology OR “mentally ill” OR “attention deficit” OR adhd OR perception* OR attitude* OR "body image" ) OR AB ( mental W1 (health OR illness* OR disease* OR disorder* OR stress* OR defect* OR instability) ) OR AB ( (behavior* OR behaviour*) N2 (disorder* OR disturbance) ) OR AB ( (academic OR school) N2 (performance OR achievement*) ) OR AB ( (social* OR peer*) N2 (accept* OR wellbeing OR "well being" OR interact*) ) OR AB ( self W1 (concept OR esteem OR confidence OR image) ))
9. (((((((((((((((((((((((((((((((((((((DE "Social Determinants of Health") OR (DE "Health Care Access" OR DE "Treatment Barriers")) OR (DE "Health Insurance" OR DE "Employee Health Insurance" OR DE "Fee for Service" OR DE "Health Maintenance Organizations" OR DE "Medicaid" OR DE "Medicare" OR DE "Underinsured (Health Insurance)" OR DE "Uninsured (Health Insurance)")) OR (DE "Health Literacy" OR DE "Mental Health Literacy")) OR (DE "Educational Attainment Level")) OR (DE "Social Inclusion")) OR (DE "Social Exclusion")) OR (DE "Social Deprivation" OR DE "Social Isolation")) OR (DE "Social Integration" OR DE "School Integration")) OR (DE "Sense of Community")) OR (DE "Belonging")) OR (DE "Loneliness")) OR (DE "Social Support" OR DE "Perceived Social Support")) OR (DE "Social Networks" OR DE "Online Social Networks")) OR (DE "Prejudice" OR DE "Religious Prejudices")) OR (DE "Social Discrimination" OR DE "Age Discrimination" OR DE "Disability Discrimination" OR DE "Employment Discrimination" OR DE "Intersectionality" OR DE "Race and Ethnic Discrimination" OR DE "Sex Discrimination" OR DE "Social Class Bias" OR DE "Weight-Based Discrimination")) OR (DE "Working Conditions" OR DE "Job Enrichment" OR DE "Noise Levels (Work Areas)" OR DE "Occupational Safety" OR DE "Telecommuting" OR DE "Work Rest Cycles" OR DE "Work Week Length" OR DE "Workday Shifts" OR DE "Working Space")) OR (DE "Incarceration" OR DE "Incarcerated")) OR (DE "Income (Economic)" OR DE "Salaries")) OR (DE "Economic Security")) OR (DE "Social Status")) OR (DE "Socioeconomic Factors" OR DE "Economic Disadvantage" OR DE "Economic Resources" OR DE "Employment Status" OR DE "Income Level" OR DE "Social Class" OR DE "Social Disadvantage" OR DE "Socioeconomic Disparities" OR DE "Socioeconomic Status")) OR (DE "Government Policy Making" OR DE "Education Policy" OR DE "Foreign Policy Making" OR DE "Law (Government)" OR DE "Laws" OR DE "Legislative Processes" OR DE "National Security" OR DE "Older Americans Act" OR DE "Peacekeeping" OR DE "Welfare Reform")) OR (DE "Employment Status" OR DE "Employability" OR DE "Employment History" OR DE "Job Loss" OR DE "Reemployment" OR DE "Retirement" OR DE "Self-Employment" OR DE "Unemployment")) OR (DE "Job Security")) OR (DE "Poverty" OR DE "Food Insecurity" OR DE "Poverty Reduction")) OR (DE "Housing" OR DE "Assisted Living" OR DE "Dormitories" OR DE "Group Homes" OR DE "Retirement Communities" OR DE "Shelters" OR DE "Transitional Housing")) OR (DE "Home Environment" OR DE "Living Arrangements")) OR (DE "Neighborhoods" OR DE "Ghettoes")) OR (DE "Crime" OR DE "Arson" OR DE "Child Abuse" OR DE "Crime Victims" OR DE "Criminal Profiling" OR DE "Criminal Record" OR DE "Cybercrime" OR DE "Driving Under the Influence" OR DE "Hate Crimes" OR DE "Human Trafficking" OR DE "Illegal Drug Distribution" OR DE "Kidnapping" OR DE "Perpetrators" OR DE "Serial Crime" OR DE "Sex Offenses" OR DE "Theft" OR DE "Vandalism" OR DE "Violent Crime")) OR (DE "Violence" OR DE "Domestic Violence" OR DE "Gender Violence" OR DE "Gun Violence" OR DE "Patient Violence" OR DE "Police Violence" OR DE "Political Violence" OR DE "School Violence" OR DE "Sexual Violence" OR DE "Violent Crime" OR DE "Virtual Violence" OR DE "Workplace Violence")) OR (DE "Transportation" OR DE "Air Transportation" OR DE "Ground Transportation" OR DE "Public Transportation" OR DE "Water Transportation")) OR (DE "Environmental Health")) OR (DE "Communication Barriers")) OR (DE "Culture (Anthropological)" OR DE "Cultural Appropriation" OR DE "Cultural Bias" OR DE "Cultural Capital" OR DE "Cultural Competence" OR DE "Cultural Deprivation" OR DE "Cultural Identity" OR DE "Cultural Sensitivity" OR DE "Folklore" OR DE "Memes" OR DE "Multiculturalism" OR DE "Popular Culture" OR DE "Society" OR DE "Subculture (Anthropological)" OR DE "Traditions" OR DE "Visual Culture")) OR (DE "Stress" OR DE "Academic Stress" OR DE "Acute Stress" OR DE "Caregiver Burden" OR DE "Chronic Stress" OR DE "Environmental Stress" OR DE "Financial Strain" OR DE "Minority Stress" OR DE "Occupational Stress" OR DE "Perceived Stress" OR DE "Physiological Stress" OR DE "Posttraumatic Stress" OR DE "Psychological Stress" OR DE "Social Stress" OR DE "Stress Reactions" OR DE "Trauma")) OR (DE "Physical Activity" OR DE "Actigraphy" OR DE "Exercise")) OR (DE "Recreation Areas" OR DE "Playgrounds" OR DE "Public Space")
10. (TI( "social determin*" OR "social factor*" OR "social health determinant*" OR sdoh OR "health structural determinant*" OR "health literacy" OR "ehealth literacy" OR "education level*" OR "level of education" OR "educational attainment" OR "educational achievement" OR "sense of belonging" OR "sense of community" OR isolation OR loneliness OR discrimination OR prejudice* OR racism OR homophobia OR biphobia OR transphobia OR "workplace condition*" OR "workplace environment" OR incarceration OR imprisonment OR prisoner* OR "economic stability" OR income OR "cost of living" OR socioeconomic* OR socio-economic* OR "social economic status" OR "public polic*" OR "government* polic*" OR poverty OR employment OR unemployment OR "occupational status" OR "job security" OR "job insecurity" OR "food security" OR "food insecurity" OR housing OR lodging* OR "living arrangement*" OR transportation OR "air quality" OR "air pollution" OR "water quality" OR "healthy food*" OR "food desert*" OR neighborhood* OR neighbourhood* OR "physical environment" OR crime OR violence OR "language barrier*" OR "communication barrier*" OR (driver* W@ health) OR doh OR "structural conflict*" OR culture OR stress OR "physical activit*" OR exercise OR parks OR "green space*" OR walkability OR "walk friendly" OR "walking friendly" OR playground* ) OR TI( (healthcare OR care OR "health services") N2 access* ) OR TI( (health OR healthcare OR "health care" OR medical OR sickness) W1 (insurance OR benefit*) ) OR TI( (civic OR social OR community OR public) W1 (participation OR engagement OR integration OR inclusion OR involvement) ) OR TI( social W1 (status OR achievement OR condition* OR protection OR polic* OR environment OR connectedness OR cohesion OR context* OR exclu* OR support* OR network*) )) OR (AB( "social determin*" OR "social factor*" OR "social health determinant*" OR sdoh OR "health structural determinant*" OR "health literacy" OR "ehealth literacy" OR "education level*" OR "level of education" OR "educational attainment" OR "educational achievement" OR "sense of belonging" OR "sense of community" OR isolation OR loneliness OR discrimination OR prejudice* OR racism OR homophobia OR biphobia OR transphobia OR "workplace condition*" OR "workplace environment" OR incarceration OR imprisonment OR prisoner* OR "economic stability" OR income OR "cost of living" OR socioeconomic* OR socio-economic* OR "social economic status" OR "public polic*" OR "government* polic*" OR poverty OR employment OR unemployment OR "occupational status" OR "job security" OR "job insecurity" OR "food security" OR "food insecurity" OR housing OR lodging* OR "living arrangement*" OR transportation OR "air quality" OR "air pollution" OR "water quality" OR "healthy food*" OR "food desert*" OR neighborhood* OR neighbourhood* OR "physical environment" OR crime OR violence OR "language barrier*" OR "communication barrier*" OR (driver* W2 health) OR doh OR "structural conflict*" OR culture OR stress OR "physical activit*" OR exercise OR parks OR "green space*" OR walkability OR "walk friendly" OR "walking friendly" OR playground* ) OR AB( (healthcare OR care OR "health services") N2 access* ) OR AB( (health OR healthcare OR "health care" OR medical OR sickness) W1 (insurance OR benefit*) ) OR AB( (civic OR social OR community OR public) W1 (participation OR engagement OR integration OR inclusion OR involvement) ) OR AB( social W1 (status OR achievement OR condition* OR protection OR polic* OR environment OR connectedness OR cohesion OR context* OR exclu* OR support* OR network*) ))
11. S7 OR S8
12. S9 OR S10
13. S3 AND S6 AND S11 AND S12

**Europe PMC**

*One-line search run in the Basic Search interface [note: due to character limits in the search interface, some terms had to be left out of this search]:*

TITLE_ABS:(child* OR pediatric* OR paediatric* OR pediatry OR adolescen* OR teen* OR youth*) AND TITLE_ABS:( stabism* OR squint OR hypertropia* OR amblyop* OR cataract* OR “lens clouding” OR “lens opacity” OR blindness OR amauros* OR “low vision” OR glaucoma* OR maculopath* OR myopi* OR nearsighted* OR oculopathy OR ophthalmo* OR astigmatism OR hyperop* OR farsighted* OR longsighted* OR macropsia* OR micropsia* OR “loss of vision” OR "visual impairment" OR "visually impaired" OR "vision impairment" OR "vision disorders" OR "eye diseases" OR "eye disorders" OR "macular degeneration" OR "retina detachment" OR "detached retina") AND TITLE_ABS:( psychologic* OR neuropsych* OR psychiatr* OR psychopathology OR “mentally ill” OR “attention deficit” OR adhd OR perception* OR attitude* OR "body image" OR "mental health" OR "mental illness" OR "mental stress" OR "mental disorders" OR "mental disease" OR "mental defect" OR "mental instability" OR "behavior disorder" OR "behaviour disorder" OR "behavior disturbance" OR "behavior disorder" OR "academic performance" OR "school performance" OR "academic achievement" OR "school achievement" OR "social acceptance" OR "social wellbeing" OR "social well being" OR "social interaction" OR "peer acceptance" OR "self concept" OR "self esteem" OR "self confidence" OR "self image") AND TITLE_ABS:( "social determinant" OR "social determinants" OR sdoh OR "social factors" OR "social health determinant" OR "health structural determinant" OR socioeconomic* OR income OR workplace OR employment OR unemployment OR education OR food OR housing OR racism OR discrimination OR prejudice OR isolation OR loneliness OR pollution OR "language barrier" OR "health literacy" OR "community engagement" OR "social engagement" OR "access to care" OR "access to healthcare" OR "access to health" OR "health insurance" OR "community involvement" OR "community participation" OR "social participation" OR "social inclusion" OR "social exclusion" OR "social support" OR "social network" OR "social status" OR "social environment" OR neighborhood* OR neighbourhood* OR lodging OR "living arrangements" OR stress OR "physical activity" OR parks OR playground* OR crime OR violence OR incarceration OR imprisonment OR prisoner OR "economic stability" OR "cost of living" OR "social economic status" OR "public policy" OR "government policy" OR poverty OR transportation OR job OR "occupational status")
